# MS3ALIGN: an efficient molecular surface aligner using the topology of surface curvature

**DOI:** 10.1186/s12859-015-0874-8

**Published:** 2016-01-12

**Authors:** Nithin Shivashankar, Sonali Patil, Amrisha Bhosle, Nagasuma Chandra, Vijay Natarajan

**Affiliations:** Department of Computer Science and Automation, Indian Institute of Science, Bangalore, 560012 India; Department of Biochemistry, Indian Institute of Science, Bangalore, 560012 India; Department of Computer Science and Automation, and Supercomputer Education and Research Centre, Indian Institute of Science, Bangalore, 560012 India

**Keywords:** Molecular alignments, Molecular surfaces

## Abstract

**Background:**

Aligning similar molecular structures is an important step in the process of bio-molecular structure and function analysis. Molecular surfaces are simple representations of molecular structure that are easily constructed from various forms of molecular data such as 3D atomic coordinates (PDB) and Electron Microscopy (EM) data.

**Methods:**

We present a Multi-Scale Morse-Smale Molecular-Surface Alignment tool, MS3ALIGN, which aligns molecular surfaces based on significant protrusions on the molecular surface. The input is a pair of molecular surfaces represented as triangle meshes. A key advantage of MS3ALIGN is computational efficiency that is achieved because it processes only a few carefully chosen protrusions on the molecular surface. Furthermore, the alignments are partial in nature and therefore allows for inexact surfaces to be aligned.

**Results:**

The method is evaluated in four settings. First, we establish performance using known alignments with varying overlap and noise values. Second, we compare the method with SurfComp, an existing surface alignment method. We show that we are able to determine alignments reported by SurfComp, as well as report relevant alignments not found by SurfComp. Third, we validate the ability of MS3ALIGN to determine alignments in the case of structurally dissimilar binding sites. Fourth, we demonstrate the ability of MS3ALIGN to align iso-surfaces derived from cryo-electron microscopy scans.

**Conclusions:**

We have presented an algorithm that aligns Molecular Surfaces based on the topology of surface curvature. A webserver and standalone software implementation of the algorithm available at http://vgl.serc.iisc.ernet.in/ms3align.

**Electronic supplementary material:**

The online version of this article (doi:10.1186/s12859-015-0874-8) contains supplementary material, which is available to authorized users.

## Introduction

Three dimensional solved crystal structures of proteins provide valuable insights regarding the function of the protein as the precise position of all functionally and structurally important residues is known. Since structure determines function, the function of an unknown protein may be determined by comparing its structure to structures of proteins whose functions are already known. Tools such as MUSTANG and DALI [1, 2], which are widely used to compare protein structures, use three-dimensional co-ordinates of atoms in the protein structures as inputs and report structural dissimilarities in terms of an RMS distance between their aligned coordinates.

Proteins that function as enzymes and transporters contain a pocket or the binding site in the structure that accommodates the substrate and cargo small molecules respectively. The arrangement of amino acid residues in the binding site often determines the specificity of a small molecule ligand towards a receptor protein. It is intuitive therefore that structurally similar ligands will bind to pockets that are structurally similar. Therefore, pocket and ligand alignments could potentially provide insights into protein function.

There exists a large number of tools that determine alignments. Common approaches for determining alignments include aligning residues [1, 3–5], secondary structures [6, 7], or molecular surfaces [8–12].

Surface based methods offer advantages in the study of protein-ligand and protein-protein interactions as they determine alignments based on the molecular surfaces at the site of interaction.

The tool MOLLOC [8] and the image-based method by Merelli et al. [11] compute alignments by comparing images of the surface from *oriented points* to determine corresponding points on surfaces. Since the number of oriented points is large, these tools are computationally intensive and require fine tuning for efficient execution. PROBIS [10] aligns proteins/nucleic acids by aligning the centroids of sub-residue level functional units (aliphatic/aromatic rings, hydrogen donor/acceptors etc) that lie close to the molecular surface. The tool SURFCOMP aligns small ligand surfaces using surface curvature along with either electrostatic potential or lipophilic potential on the molecular surfaces. SURFCOMP identifies local maxima of these scalar fields as features and attempts to align them across pairs of surfaces. The tool PBSALIGN aligns protein-protein interaction surfaces using an approach similar to SURFCOMP with a few key differences. It defines feature points as surface points that are closest to *C*_*α*_ atoms and computes a feature vector comprising of principal curvatures and statistics of electrostatic potential and hydrophobicity near each feature point. Zhang and Hebert [13] propose the use of harmonic maps for surface matching. Their two step approach first constructs a bijective map from a disc-like surface patch to a planar disc. It next employs a standard image matching algorithm to register two planar discs and thus aligns the surfaces due to the bijection of the map. The method however assumes that the patches have a disk-like shape and depends on an initial construction of a map from the boundary of the surface patch to the planar disc. Furthermore, the method is computationally expensive as it involves a least squares minimization for each patch.

Cosgrove et al. [14] present a surface curvature based method similar to SURFCOMP that is applicable for small ligands. It differs from SURFCOMP primarily in the construction of surface patches to elicit landmark points.

Goldman et al. [15] describe a surface curvature based matching method that recognizes similar patches by fitting least squares quadric surfaces and comparing their curvature values. These quadrics are located at the centroid of each surface patch of the solvent excluded surface (SES). Again, the method is restricted to small ligands and cannot be easily scaled to large molecules. Furthermore, the choice of the centroids is driven by the computation of the SES. Exner et al. [16] present an approach based on fuzzy sets where scalar quantities, such as curvature and electrostatic potential, are translated to linguistic variable classes (for example, convex, flat, or concave regions). Then, a normalized comparison measure is used to construct regions of similar linguistic semantics. These semantically coherent regions are coupled with artificial neural networks to automatically find active sites in proteins. Baum and Hege [17] present a SES alignment method that aligns patches generated by an approximation of geodesic Voronoi diagrams. Furthermore, they incorporate semantic similarity in ranking their alignments by introducing additional points in regions of similar function (for example, a donor or receptor region).

### Summary of results

We present MS3ALIGN, a Multi-Scale, Morse-Smale, Molecular Surface aligner. The tool MS3ALIGN begins by computing mean curvature at all points on the surface and then segmenting significant protrusions. Segmentation is performed by a topological analysis of the surface mean curvature using the Morse-Smale complex.

Correspondences between pairs of protrusions on either surface are then established using a two-step procedure. First, a multi-scale curvature descriptor is computed for each protrusion followed by neighbor identification in the descriptor space.

These correspondences are then grouped together into maximal sets. Each maximal set is used to compute a rigid body transformation that aligns the first surface to the second surface. These alignments are evaluated and ordered using a distance measure that is based on the RMS distance between surfaces and the corresponding area fraction.

A key benefit of MS3ALIGN is its controlled generation of landmark points based on desired feature size. The landmarks are crucial for the efficient execution of the subsequent alignment steps (see “Parameter selection” section for details on tuning parameters for landmark generation). This is a primary difference between MS3ALIGN and various other methods that employ surface curvature as a primary geometric descriptor to extract landmark points [9, 12, 14, 15].

Similar to the other surface geometry based methods, MS3ALIGN is applicable even in situations when specific physico chemical properties or resolved atomic co-ordinates are not directly available. For example, the electrostatic potential or the resolved atomic structure may not be available for Cryo-Electron-Microscopy data. We describe experimental results where surfaces generated from both, atom location data in the PDB and density maps in the Electron-Microscopy data bank, are aligned.

Our method may be viewed as an improvement of SURFCOMP [9], and hence we perform a detailed comparison with their results. We validate MS3ALIGN using surface representations of ligands and their site of interaction. Specifically, we use the POCKETMATCH [18] tool to quantify structural variation between various interaction sites and compare their alignments obtained using MS3ALIGN. In this experiment, we also demonstrate the benefit of visual analysis where we visually validate results.

## Background

We briefly review the necessary background relevant for this paper. We begin by discussing the mathematical notion of curvature and algorithms to compute them for triangular meshes. We then discuss the Morse-Smale complex and its simplification.

### Mean curvature

**Fig. 1:**
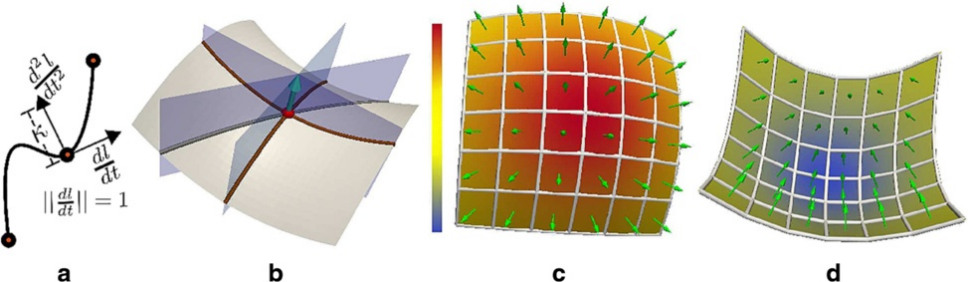
**a** Curvature *κ* of a regular curve at a point is defined as the magnitude of the second differential at that point. **b** The principal curvatures of a point *x* on a smooth surface is defined as the maximal *κ*_1_ and minimal *κ*_2_ curvature of regular curves formed by intersecting planes rotated about the surface normal *n*_*x*_ with the surface. Mean curvature is defined as (*κ*_1_+*κ*_2_)/2. Mean curvature is **c** high at convex regions **d** and low at concave regions. Green arrows depict surfaces normals at respective surface points

A *regular curve* is defined as a twice differentiable function $l(t):\mathbb {I} \rightarrow \mathbb {R}^{3} $ from the unit interval to 3D space where the magnitude of the tangent equals one, $||\frac {dl}{dt}|| = 1$ [19]. The *curvature* at a point *t* is defined as the magnitude of the second differential at *t*, $\kappa (t) = ||\frac {d^{2}l}{dt^{2}}||$ (see Fig. 1a).

A family of regular curves through a point *x* on a surface $\mathbb {S}$ is defined by a collection of planes that contain the surface normal *n*_*x*_ at the point *x* and the point itself (see Fig. 1b). Each of these curves has an associated curvature *κ*. For curves on surfaces, one associates a sign with the curvatures given by the dot product of the surface normal and the second differential of the regular curve. The curves with maximum and minimum curvature (with sign) are referred to as the *principal curvatures* at *x* and are denoted by *κ*_1_ and *κ*_2_ respectively.

The *mean curvature* at a point *x*, *H*(*x*) is the mean of *κ*_1_ and *κ*_2_ i.e. *H*(*x*)=(*κ*_1_+*κ*_2_)/2. Convex surfaces have positive *κ*_1_ and *κ*_2_ and therefore have positive mean curvature, whereas concave surfaces have negative mean curvature (see Fig. 1c and d).

### Morse-Smale complexes

**Fig. 2:**
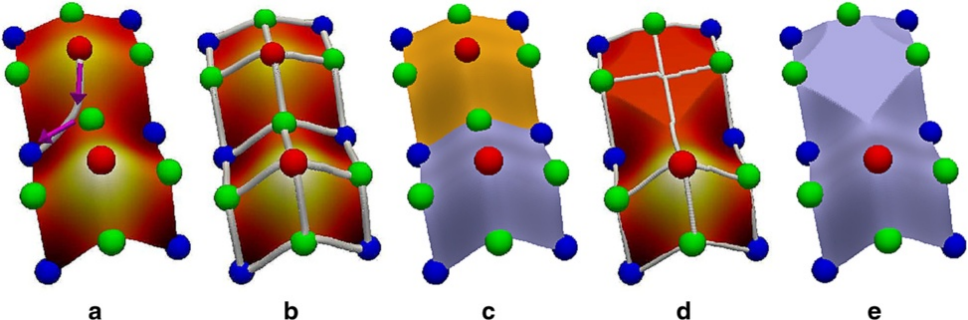
**a** Three types of criticalities in a Morse function defined on a smooth surface: maxima (red spheres), saddles (green spheres), and minima (blue spheres). An integral line originates at a minimum and terminates at a maximum. **b** The Morse-Smale complex is a partition of the domain into regions whose integral lines share a common source and destination critical point. The combinatorial structure of the Morse-Smale complex is a graph whose arcs are incident on an maximum-saddle or a saddle-minimum pair. **c** The descending manifold of a maximum is the region defined by the integral lines that converge to it. **d** The Morse-Smale complex may be simplified by local modification of the function resulting in the cancellation of a pair of critical points that are adjacent in the combinatorial structure. **e** After simplification, the descending manifold of the maximum that is adjacent to the canceled saddle expands to include the descending manifold of the canceled maximum

The Morse-Smale complex is a topological data structure that is defined based on the gradients of a scalar function. Given a surface $\mathbb {S}$ and a scalar function $f:\mathbb {S} \rightarrow \mathbb {R}$, the gradient of *f* at a point *x* is defined by the partial derivative with respect to the local coordinates at *x* i.e. ∇*f*(*x*)=(*∂f*/*∂u*,*∂f*/*∂v*). A *critical point* is a point *x* in $\mathbb {S}$ whose gradient is zero. A critical point is called *non-degenerate*, if the *Hessian*, equal to the matrix of second order partial derivatives of *f*, is non-singular. The function *f* is said to be a *Morse function* if all its critical points are non-degenerate. The *index* of a critical point is the number of negative eigenvalues of the *hessian*. In 2D, there exist three types of critical points namely the maximum, saddle, and minimum. An *integral line* is a curve embedded in $\mathbb {S}$ whose tangent aligns with the gradient at every point (see Fig. 2a). Integral lines originate and terminate at critical points. The Morse-Smale complex is a partition based on the source and the destination of the integral lines [20] (see Fig. 2b). The combinatorial structure of the Morse-Smale complex is a graph whose nodes correspond to critical points, and edges or arcs exist between the nodes if there exists an integral line that connects the corresponding critical points and their indices differ by one (see Fig. 2b). The descending manifold of a maximum consists of integral lines that converge to it (see Fig. 2c). Similarly, the ascending manifold of a minimum consists of integral lines that diverge from it. To deal with noisy functions, the Morse-Smale complex may be simplified by the process of topological simplification, which locally modifies the function to remove saddle-minimum or saddle-maximum pairs (see Fig. 2d). After simplification the descending manifold of a canceled maximum is merged with the descending manifold of the maximum connected to the canceled saddle (see Fig. 2e). It is often sufficient to directly obtain a simplified Morse-Smale complex instead of modifying the function in order to extract and analyze features. Ordering of simplification pairs is crucial in determining the resulting structure. The theory of topological persistence is a common choice for ordering of pairs for simplification [21]. Persistence-directed simplification iteratively eliminates arcs in the combinatorial structure with smallest absolute difference in function value and terminates when a specified simplification threshold is achieved.

## Methods

**Fig. 3:**
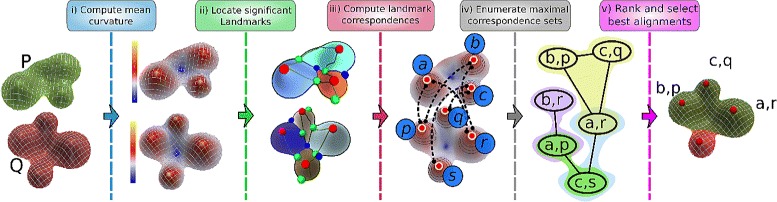
MS3ALIGN comprises of five stages. *P* and *Q* are input surfaces represented as triangle meshes. **i** Mean curvature is computed for both surfaces. **ii** Significant landmarks are extracted by first computing the Morse-Smale complex of the curvature field and then simplifying the Morse-Smale complex using topological persistence. The maxima that survive simplification (red spheres) are used as landmark points. **iii** Correspondences between landmark points on either surface is established by comparing the multi-scale curvature vectors. **iv** A graph is constructed where each landmark correspondence is considered as a node, and edges are placed between nodes if they satisfy inequalities (1) and (2). Each maximal clique in this graph generates a maximal correspondence set. **v** Each maximal correspondence set is evaluated using the measure given by (3). Those evaluating to the smallest values are ranked as the best alignments

In this section we describe the design of MS3ALIGN in detail. The tool MS3ALIGN comprises of multiple stages where the primary input is the two surfaces represented as triangle meshes. Figure 3 depicts the various stages. In the following sections each stage is explained in detail.

### Curvature computation

The mean curvature at every vertex of the two input surfaces *P* and *Q* is computed in the first stage.

There exist many algorithms to estimate the mean curvature of triangle meshes. We use the algorithm based on the theory of normal cycles by Cohen-Steiner et al. [22]. We choose this approach for two reasons. First, this algorithm guarantees linear convergence to the curvature of a smooth surface, with a sufficiently well sampled set of points.

Second, the definition of the curvature is based on averaging the curvature tensor over a neighborhood of size *R*_*c*_ on the triangle mesh. This allows for a smoother estimate of the mean curvature where meshing artifacts are overcome by the averaging operation. Good selection of the neighborhood size *R*_*c*_ for curvature computation is crucial to avoid isotropy issues introduced by mesh discretization. For computing a smooth curvature estimate at each vertex, we find that the neighborhood size *R*_*c*_ needs to span at least two rings of vertices. The maxima of mean curvature correspond to the protrusions on each surface patch.

### Landmark extraction

In the second stage, landmark points, representing significant protrusions of both surfaces *P* and *Q*, are identified from the maxima of the mean curvature scalar field. However, several maxima may correspond to regions with low mean curvature. Significant protrusions are identified by a topological analysis using the Morse-Smale complex of the mean curvature field. Segmenting molecular surfaces using the Morse-Smale complex has been reported earlier [23, 24] using the Connolly function [25], which is related to the surface curvature.

The Morse-Smale complex of the mean curvature field is first computed [26] and then simplified by iteratively canceling insignificant maxima using the topological cancellation procedure.

The significance of a maximum is determined by the notion of *topological persistence* [21] where each maximum is paired with a saddle critical point. The measure of significance of a maximum, referred to as its *persistence*, is the absolute difference in mean curvature value of the maximum and its paired saddle critical point. Thus, maxima are eliminated in increasing order of persistence up to a given threshold, denoted by *T*_*s*_. The threshold *T*_*s*_ is specified as a fraction of the average of the mean curvature at all local maxima.

### Landmark correspondences

In the third stage, correspondences between pairs of landmark points on either surface *P* and *Q* is established. This is done by analysis of the curvature at multiple scales. The mean curvature computed using a neighborhood size *R*_*c*_ gives an estimate of curvature at that scale. We compute mean curvature at multiple scales at each landmark point **p**∈*P* and **q**∈*Q*. We use 15 uniformly sampled curvature scales from the interval [*R*_*c*_,2*R*_*c*_]. Landmark points **p**∈*P* and **q**∈*Q* are declared as a corresponding pair (**p**,**q**) if the absolute difference between their mean curvatures at every scale is bounded by a threshold *T*_*ms*_. The threshold *T*_*ms*_ is specified as a fraction similar to *T*_*s*_.

### Maximal correspondence sets

In the fourth stage, correspondences between landmark points in *P* and *Q* are collected into maximal sets of correspondences. A maximal set $\mathbb {C} := \left \{(\mathbf {p}_{1}, \allowbreak \mathbf {q}_{1}), \allowbreak (\mathbf {p}_{2},\mathbf {q}_{2}), \allowbreak \ldots,\allowbreak (\mathbf {p}_{n},\mathbf {q}_{n})\right \}$ is constructed so that for each pair of correspondences (**p**_*i*_,**q**_*i*_) and (**p**_*j*_,**q**_*j*_), the two landmark points **p**_*i*_ and **p**_*j*_ in *P* have relative pairwise geometric properties similar to that of the two landmark points **q**_*i*_ and **q**_*j*_ in *Q*. We use two geometric properties to establish relative pairwise similarity. First, we ensure that the absolute difference between the distances of the two landmarks on either surface is less than a threshold *T*_*mrd*_, referred to as the maximum relative distance threshold. Second, we ensure that the absolute difference in the angles between the surface normals of two landmarks on either surface is less than *π*/2. In other words, for a given maximal set $\mathbb {C} := \left \{(\mathbf {p}_{1}, \allowbreak \mathbf {q}_{1}), \allowbreak (\mathbf {p}_{2},\mathbf {q}_{2}), \allowbreak \ldots,\allowbreak (\mathbf {p}_{n},\mathbf {q}_{n})\right \}$, we ensure that

(1) $$ {\vert} \Vert \mathbf{p}_{i}-\mathbf{p}_{j}\Vert - \Vert \mathbf{q}_{i}-\mathbf{q}_{j}\Vert \vert < T_{mrd}   $$

(2) $$ {\vert} \cos^{-1}(N(\mathbf{p}_{i}).N(\mathbf{p}_{j})) - \cos^{-1}(N(\mathbf{q}_{i}).N(\mathbf{q}_{j})) {\vert}< \raisebox{1ex}{$\pi$}\left/ \raisebox{-1ex}{$2$}\right.   $$

for all $(\mathbf {p}_{i},\mathbf {q}_{i}), (\mathbf {p}_{j},\mathbf {q}_{j}) \in \mathbb {C}$, where *N*(**p**) represents the surface normal at point **p**. The constructed sets are maximal in the sense that no other correspondence may be added without violating conditions (1) and (2).

Finding maximal correspondence sets may be recast as the problem of enumerating maximal cliques in graphs. The nodes of the graph are correspondences between landmark points (**p**,**q**). Edges exist between pairs of correspondences (**p**,**q**) and (*p*^′^,*q*^′^) if they satisfy conditions (1) and (2) and if **p**≠*p*^′^ as well as **q**≠*q*^′^. Maximal correspondence sets are found by enumerating maximal cliques from this graph.

We use the greedy pivot heuristic modification of the Bron-Kerbosch algorithm [27] by Koch [28, 29] to enumerate maximal cliques in this graph. This modification exhibits near linear complexity in the number of maximal cliques for most graphs.

Indeed, the total number of maximal correspondence sets may be exponential in the number of landmark points. The number of maximal correspondence sets depends on the tolerance for positional uncertainty of the landmark points, captured by *T*_*mrd*_, as well as the number of correspondences, captured by *T*_*ms*_. Choosing a very small *T*_*ms*_ and *T*_*mrd*_ causes the algorithm to demand near exact matches in the mean curvature as well as the relative positions of the landmarks, whereas higher values allow larger variations.

### Surface alignment

In the final stage, each maximal correspondence set is first used to determine a rigid body transformation **(R, t)** from *P* to *Q* via a least squares minimization [30].

Next, for each correspondence set ${\mathbb {C}} := \left \{(\mathbf {p}_{1}, \mathbf {q}_{1})\right., $ (**p**_2_,**q**_2_),…,(**p**_*n*_,**q**_*n*_)}, we compute a measure $D_{P,Q}(\mathbb {C})$ that quantifies the error of the transformation (**R,t**), given by

(3) $$ \begin{aligned} D'_{P,Q}(\mathbb{C}) &:= \frac{\sqrt{\frac{\sum_{i=1}^{n} A_{P}(\mathbf{p}_{i}) \Vert (\mathbf{R}\mathbf{p}_{i} + \mathbf{t}) - \mathbf{q}_{i} \Vert^{2}}{A_{P}(\mathbb{C})}}}{
\raisebox{1ex}{$A_{P}(\mathbb{C})$}\left/ \raisebox{-1ex}{$A_{P}$}\right.} \\ D_{P,Q}(\mathbb{C}) &:= \min \left\{D'_{P,Q}(\mathbb{C}), D'_{Q,P}(\mathbb{C})\right\} \end{aligned}   $$

Here, *A*_*P*_(**p**_*i*_) is the area of the descending manifold of **p**_*i*_, $A_{P}(\mathbb {C})$ is the total area of all landmarks of *P* in $\mathbb {C}$ i.e. $A_{P}(\mathbb {C}) := \sum _{i=1}^{n} A(\mathbf {p}_{i})$, and *A*_*P*_ is the area of *P*. Also, $D_{P,Q}(\mathbb {C})$ is a symmetric version of $D^{\prime }_{P,Q}(\mathbb {C})$, where $D^{\prime }_{Q,P}(\mathbb {C})$ inverts the roles of *P* and *Q* in $D^{\prime }_{P,Q}(\mathbb {C})$ with the exception that the transformation (**R,t**) is again applied only to landmarks in *P*.

The numerator of $D^{\prime }_{P,Q}(\mathbb {C})$ in Eq. (3) represents a coarse approximation of the RMS distance between the matching portions of both surfaces. The denominator is the fraction of the area of descending manifolds of all landmark points of *P* in $\mathbb {C}$ with respect to the total area of *P*. Due to the denominator, correspondence sets having larger area fractions are preferred.

Maximal correspondence sets that result in the least values of this measure are reported along with their transformations. Computing this measure is efficient even with a large numbers of maximal correspondence sets since it only requires landmarks and not all points on the surface. It is possible for some spurious correspondence sets consisting of a few landmarks to align nearly perfectly. Hence, we consider only those correspondence sets so that the area of the corresponding regions of either surface is at least 15 *%* of its total area.

### Parameter selection

The four parameters *R*_*c*_, *T*_*s*_, *T*_*ms*_ and *T*_*mrd*_ control the performance of the MS3ALIGN algorithm. While the parameters *R*_*c*_ and *T*_*s*_ control the quality of the landmarks, the parameters *T*_*mrd*_ and *T*_*ms*_ control how these landmarks are aligned. The expected size/scale of features must have direct bearing on the choice of *R*_*c*_ and *T*_*mrd*_ because they are distance values (specified in Å). Hence, this choice depends on the dataset being studied. By default, we assume *T*_*mrd*_ to be equal to *R*_*c*_. If this results in an insufficient number of landmark alignments, then *T*_*mrd*_ may be set to a higher value. Conversely, if we expect or desire less relative movement of feature points, then it is set o a lower value. The parameters *T*_*s*_ and *T*_*ms*_ are specified in a normalized scale of positive curvature values. Hence, we explicitly conducted performance experiments (“Performance analysis” section) to determine good choices of these parameters. Based on these experiments, we determine that *T*_*s*_=0.1 is a good choice. By default, we assume *T*_*ms*_ to be equal to *T*_*s*_. Again, in case there are insufficient number of landmark alignments, *T*_*ms*_ may be set to a higher value. Conversely, if there are fewer or larger number of feature points, *T*_*s*_ may be lowered or raised as desired. However, changing the value drastically will result in either oversimplification or undersimplification, both of which results in poor quality of feature points.

**Fig. 4:**
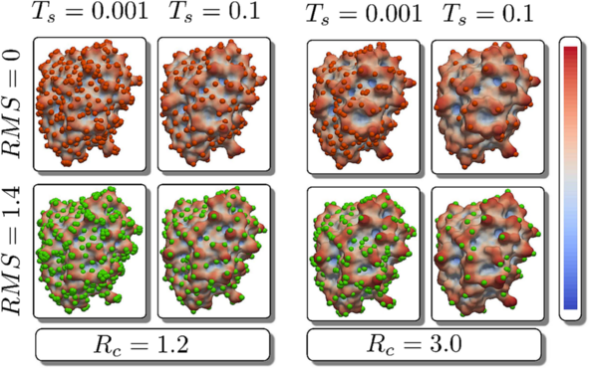
Comparing the influence of parameters in the generation of landmark points

To illustrate the consequence of these choices, we present Fig. 4. The two panels show landmarks computed for the molecule with PDB ID 4*gh*7 with *R*_*c*_ set to 1.2 and 3.0, respectively. The columns in each panel show the landmark points for values of *T*_*s*_ equal to 0.001 and 0.1. Each row shows the landmarks on the surfaces without noise and with noise (*RMSD* 1.4 Å) added to the atom locations. The mean-curvature computed is shown using a color map. Comparing two panels directly, the effect of *R*_*c*_ is evident in the density of the landmarks. The choice of *R*_*c*_=1.2 is inappropriate here as significant protrusions are not captured effectively. Raising the simplification threshold to *T*_*s*_=0.1 does not resolve the issue. Furthermore, with the addition of noise, the corresponding change in landmarks is hard to distinguish. In contrast, in the right panel where *R*_*c*_=3.0, landmarks representing significant protrusions are retained across simplification thresholds of 0.001 and 0.1 Furthermore, it is possible to visually establish the correspondences between the landmarks across the noiseless and noisy versions, particularly with *T*_*s*_ is set to 0.1.

## Results and discussion

In this section we discuss our evaluation of MS3ALIGN. We begin with a discussion on the molecular surfaces used in our experiments (“Molecular surfaces” section). In the first experiment (“Performance analysis” section), we evaluate its performance under conditions of noise, partial overlap, and running times using a random set of 20 proteins from the PDB [31]. In the second experiment (“A comparison with SURFCOMP” section), we compare MS3ALIGN with the results discussed in the evaluation of SURFCOMP [9]. As the MOLLOC web-server [8] and the code from [11] is unavailable, we could not compare our results with theirs. In the third experiment (“A validation using POCKETMATCH and PYMOL” section), we validate the alignments of binding sites computed by MS3ALIGN comparing them with those generated using PyMol [32]. Here, we quantify the structural variation of binding sites using POCKETMATCH [18].

In the fourth experiment (“Aligning electron microscopy iso-surfaces” section), we use MS3ALIGN to compute alignments of iso-surfaces extracted from cryo-electron microscopy scans.

### Molecular surfaces

In our experiments, we use three types of surfaces extracted from proteins/ligands obtained from the PDB [31] which are built upon the van der Waals molecular model. The first type is the *molecular skin surface* (“Skin surface” section) [33]. The second type of surface used is the *ligand surface*, which is representative of the ligand interacting with the protein in a protein-ligand interaction obtained from the PDB [31] (“Ligand surface” section). The third kind of surface used is the *pocket surface* (“Pocket surface” section). This is representative of the surface of interaction of the protein with the ligand. Additionally, we generate partially overlapping surfaces by cutting skin surfaces to generate pairs of surfaces with approximately known overlap fractions (“Partially overlapping surfaces” section).

#### Skin surface

The molecular skin surface is a mathematically robust surface model that is similar in geometry to the solvent excluded surface. The molecular skin surface of a protein is computed in two steps. First, its atomic locations are augmented with the van der Waals radii after adding missing hydrogen atoms using the PDB2PQR tool [34]. Next, the skin surface is extracted using the NANOSHAPER tool [35] using the atomic locations and radii as input.

#### Ligand surface

The ligand surface is computed in three steps. First, the CHIMERA [36] program is used to extract the molecular structure of the ligand from the protein’s PDB file. Then, together with the protein’s PDB file, the molecular structure of the ligand is used to determine atomic positions and radii of the ligand using the PDB2PQR tool. Finally, the ligand position and radii data is used to compute the skin surface of the ligand using NANOSHAPER.

#### Pocket surface

The pocket surface is extracted as a subset of the molecular skin surface of the protein. This subset is the part of the surface that belongs to the residues (within 4.5*Å*) that interact with the ligand. These residues are referred to as the *pocket residues* or just the *pocket*. A subset of the molecular surface is extracted so that all vertices of the subset are within the van der Waals sphere of at least one of the atoms of the pocket. An additional 0.5*Å* is added to the van der Waals sphere to account for the possible error introduced when extracting the skin surface, because NANOSHAPER uses a structured grid with edge length 0.5*Å*. This subset surface may be disconnected and/or contain holes. This topological noise is repaired using a variant of the dilation-erosion operation applied to triangle meshes [37]. The radius for both steps is set to 1.2*Å*, the radius of the hydrogen atom.

#### Partially overlapping surfaces

**Fig. 5:**
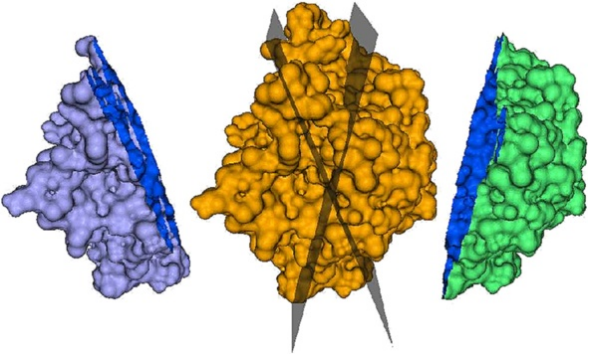
Pairs of surfaces which overlap with each other with an approximately known fraction of area of each other are generated by cutting the molecular skin surface using a pair of planes. Here, the skin surface of 4j2m (orange, center) is cut by two planes into two pieces (violet on the left and green on the right) such that 20 % of the area of either overlaps with the other

In this section, we describe in detail our approach for the generation of overlapping skin surface pairs with approximately known overlap fractions. Each skin surface is split by a pair of planes into two surfaces such that they overlap with each other with an approximately known overlap fraction (see Fig. 5). Pairs of surfaces are generated for overlap fractions of approximately 20, 40, 60, and 80 *%* of each other. To do this, first, a coordinate system about the centroid of the molecule is constructed from the principal components of the centroid subtracted positions of the surface’s vertices [38]. The plane containing the first two principal components is rotated about the second component by 0, *π*/5, 2*π*/5, 3*π*/5 and 4*π*/5 radians to obtain five planes, each of which slice the surface into two pieces. The piece on the right of each plain is retained to generate five *partial* surfaces. The first partial surface overlaps with the other four partial surface with approximately 20, 40, 60, and 80 *%* of its area.

### Performance analysis

In this experiment, we study three aspects of MS3ALIGN. First, we study its ability to determine correct alignments in the presence of noise. Second, we study its ability to detect alignments in the presence of partial overlaps. Finally, we study the runtime performance of MS3ALIGN. We use twenty structurally different proteins from the PDB [31] having 1500–3000 atoms.

We set the parameter *R*_*c*_=3*Å*. This enables landmarks to be located on protrusions of 2–4 atoms, which is typical of groups such as ammonium, hydroxy, and methyl that are close to the surface. We set the parameter *T*_*mrd*_=1*Å*. Alignments are studied with varying choices of the *T*_*s*_ parameter. The *T*_*ms*_ parameter is set to be equal to the *T*_*s*_ parameter.

We now study performance of MS3ALIGN in the presence of noise. Noise is introduced by adding standard Gaussian noise of known variance to all atom locations of the protein. The molecular skin surface of this perturbed molecule is used as the noisy version of the surface. Multiple such noisy versions of the skin surface are generated by adding increasing levels of noise. The level of noise is quantified by computing the RMS distance of all atoms from their original position to their position after adding noise. We compute the RMS distance between the two surfaces by mapping each vertex of the original surface to the closest vertex in the noisy surface after alignment.

**Fig. 6:**
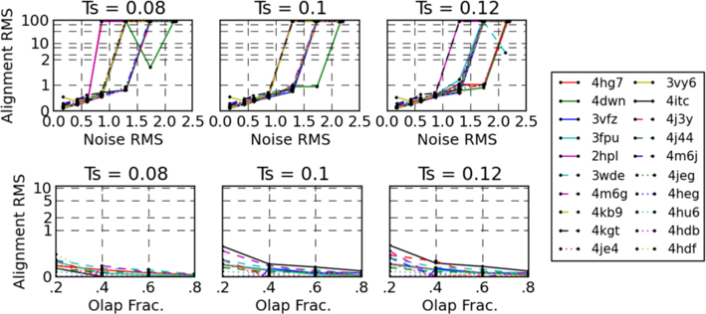
*(top-row)* RMS distance between skin surfaces of various molecules after each surface is aligned with noisy versions of itself shown for *T*_*s*_=0.08, 0.1 and 0.12 respectively. The y-axis is log-scaled beyond 2*Å*. *(bottom-row)* RMS distance between a subset of the skin surface and four other surfaces that it partially overlaps with. The y-axis is log-scaled beyond 1*Å*

We conclude that the alignment is successful if the RMS distance is within 2*Å*. The top row of Fig. 6 shows three graphs for respective simplification threshold *T*_*s*_ values of 0.08, 0.10, and 0.12.

With a *T*_*s*_ threshold of 0.1, we observe that surfaces align with RMS distance approximately equal to the RMS distance between the noisy and noiseless surfaces up to 1*Å*. We also observe that most alignments fail after the introduced noise causes RMS distance between surfaces to be more than 1*Å*. A primary reason for this is the choice of the maximum relative distance threshold *T*_*mrd*_=1Å, which specifies the amount acceptable relative movement of the landmark points.

We next study the ability of MS3ALIGN to detect alignments in the presence of partial overlaps. Five partially overlapping surfaces are generated as subsets of the skin surface. The first surface is generated by a cut plane that partitions the skin surface. The remaining surfaces are also generated by rotated cut planes such that the overlap fractions with the first surface is approximately 20, 40, 60, and 80 *%* respectively. In other words, the first and second partial surfaces intersect in approximately 20 *%* of the area of each other, the first and third intersect in approximately 40 *%* of the areas of each other, and so on. Since we already know the transformation that aligns the first partial surface with the others, namely the identity transformation, we study the RMS distance from the first partial surface after applying the the alignment transformation determined by MS3ALIGN. The bottom row of Fig. 6 shows three graphs for respective simplification threshold *T*_*s*_ values of 0.08, 0.10, and 0.12. The alignment RMS distance of the first partial surface is mapped to the y-axis and the overlap fractions with the remaining four partial surfaces is mapped to the x-axis. We find that, in all cases, alignments were successfully determined with RMS distance less than 1*Å*.

**Fig. 7:**
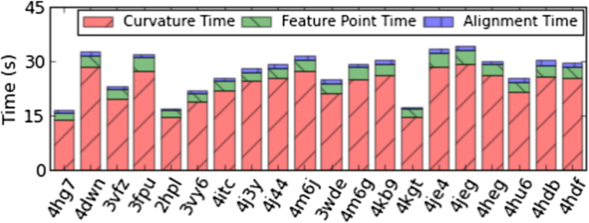
Stacked bar graph showing breakup of MS3ALIGN run-times for the surfaces used in the partial overlap study. The time taken for alignment (stages *iii*−*v*) is significantly lesser than time taken for the first and second stage

Next, we present the runtime breakup of the various stages of MS3ALIGN in Fig. 7. We conducted our experiments on a HP xw7700 workstation with an Intel(R) Xeon(R) CPU E5405 2.00 GHz dual quad-core processor and 8 GB of RAM. For efficient computation, the tool contains OpenMP directives which can be optionally enabled to leverage multi-core computation. We disable it for this experiment to run the tool on a single core, thereby enabling us to compare with other sequential methods. Fig. 7 presents the running times for the partial overlap experiment discussed above for a simplification threshold parameter *T*_*s*_ value of 0.1. We note from the figure that the alignment time is consistently under 1 s for each set of four alignments. In comparison, SURFCOMP takes about 75 s (± 15 s) for each comparison for their small ligand datasets using a 2.4 GHz Intel Xeon Processor. Baum and Hege [17] report a runtime of 5–25 s on a 3.0 GHz Intel Xeon processor for comparison of one ligand with six others. Merelli et al. [11] report that their method takes between a few seconds to about 10 min for a good match of a pair of surfaces on an Intel Dual Core machine. Angaran et al. report that MOLOC takes between few seconds to minutes for medium sized binding surface areas.

### A comparison with SURFCOMP

In this experiment, we compare MS3ALIGN with the SURFCOMP tool [9]. The authors of SURFCOMP validate using the ligand surfaces from two datasets. The first dataset consists of thermolysin inhibitor ligands of two kinds, the first containing tryptophan and the second with an aliphatic residue at the C-terminal end. The second dataset consists of ligands bound with the Dihydrofolate Reductase (DHFR) enzyme.

In both experiments, surfaces obtained from ligand molecules are aligned. Hofbauer et al. consider two types of physico-chemical properties on molecular surfaces to determine alignments.

Since they conclude that electrostatic potential (ESP) results in better alignments, we only compare against these alignments. The chemical structures of the ligands are presented in the appendix. For these experiments, the *R*_*c*_ parameter is set to 1.2*Å* since we wish to study alignments at the scale of a single atom. The *T*_*s*_ parameter is set to 0.06 for the first dataset and 0.1 for the second dataset. The *T*_*ms*_ parameter is set to 0.09 and 0.15 respectively. The *T*_*mrd*_ parameter is set equal to *R*_*c*_. In the following paragraphs, we discuss the alignments determined by MS3ALIGN for each of the datasets in detail and compare them with SURFCOMP.

In the thermolysin dataset, SURFCOMP compares eight thermolysin inhibitor ligands considered in two groups. The first group consists of ligands from PDBs 1THL, 1TLP, 1TMN and 3TMN. In our experiment, we were unable to extract the ligand structure from 3TMN because of a failure in the PDB2PQR tool which is used in a preprocessing step to compute the molecular skin surface. Hence, we remove 3TMN from this list. The second group consists of ligands from PDBs 4TLN, 5TLN, 5TMN, and 6TMN. For consistency of labeling of datasets with respect to Hofbauer et al., we use the PDB id to reference the ligand considered.

**Fig. 8:**
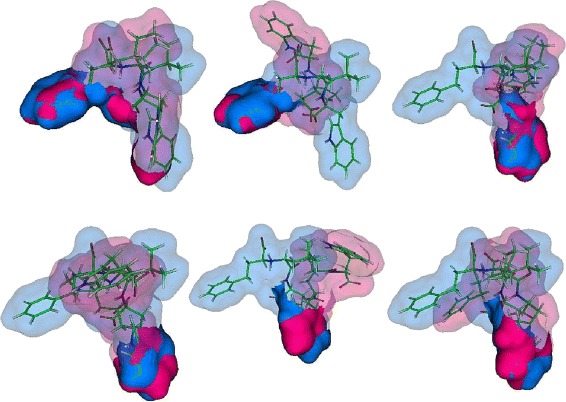
Alignment of 1THL with 1TMN exhibits multiple partial alignments. In all figures 1TMN is fixed (blue) and 1THL (pink) is transformed. *(Top Left and Center)*: The two mirrored alignments of the aromatic rings in 1THL and 1TMN are detected as the best two alignments. *(Top Right and Bottom Left)*: The two mirrored alignments of the benzyl ring of 1THL with the hetero-cycle of tryptophan in 1TMN are detected third and fourth best alignments. *(Bottom Center)*: A variant of the the alignment of the aromatic ring in 1THL with the other parts of the penta-cycle of tryptophan in 1TMN. *(Bottom Right)*: Alignment of the tryptophan parts of both ligands

**Fig. 9:**
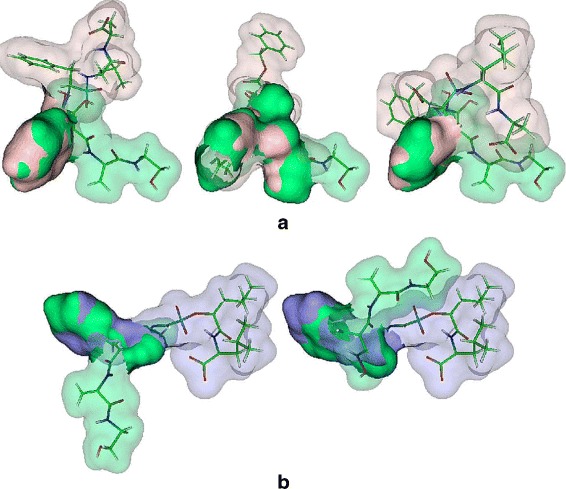
Alignments of the ligand surfaces found by MS3ALIGN and not SURFCOMP for the thermolysin inhibitor dataset. The aligning portions of the surface are shown as opaque and the rest is shown with transparency. A stick representation of the ligands is also shown. **a** The best three alignments of the 4TMN’s 0PK ligand (light pink) with 5TLN’s BAN ligand (green) aligns the two aromatic rings of 0PK with those of BAN. **b** The best two results of 6TMN’s 0PJ ligand (dark blue) with the 5TLN’s BAN(green) show alignment of aromatic rings from both ligands

**Fig. 10:**
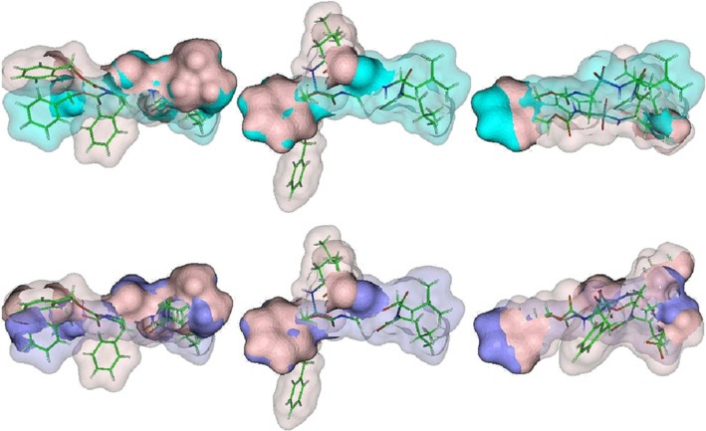
Alignments of the 4TMN’s 0PK ligand (light pink) with: *(top row)* 5TMN’s 0PJ ligand (cyan). *(bottom row)* 6TMN’s 0PK ligand (purple). The alignment of 0PK in 5TMN with BAN in 5TLN is shown in Fig. 9 a

We were able to determine the same alignments as SURFCOMP for the first group. However, in the case of 1THL-1TMN our sixth best alignment corresponds to the SURFCOMP’s best alignment. For the same pair 1THL-1TMN, SURFCOMP reports an alignment of the tryptophan moiety of both ligands. MS3ALIGN reports it as the sixth best alignment with other meaningful alignments being detected as better. Figure 8 shows the other alignments detected for the pair 1THL-1TMN. From the visualization, we confirm the alignments of similar chemical moieties (see caption of Fig. 8 for details). Also, Hofbauer et al. state that they were not able to determine relevant alignments of the ligand in 5TLN with the other ligands. Figure 9 show the best alignments of the ligand in 5TLN with the ligands in 4TMN and 5TMN using MS3ALIGN. Since the ligand 0PI (6TMN) is structurally very similar to the ligand 0PJ (5TMN), the same alignments with respect to other ligands were also found, and thus images of these alignments are omitted. Thus, we find that MS3ALIGN aligns relevant portions of the surfaces of the ligand BAN in 5TLN with all the other considered ligands. For many pairs, we determine partial alignments of other substructures. Figure 10 presents the additional alignments of 4TMN with the others in its group.

In the DHFR dataset, alignments of surfaces of four ligands interacting with DHFR are analyzed. The ligands used are Folic acid (FOL), Methotrexate (MTX), Trimethoprim (TMP), and BR-WR99210 (WRB) from PDBs 1DHF, 1DF7, 1DG5 and 1DG7 respectively.

**Fig. 11:**
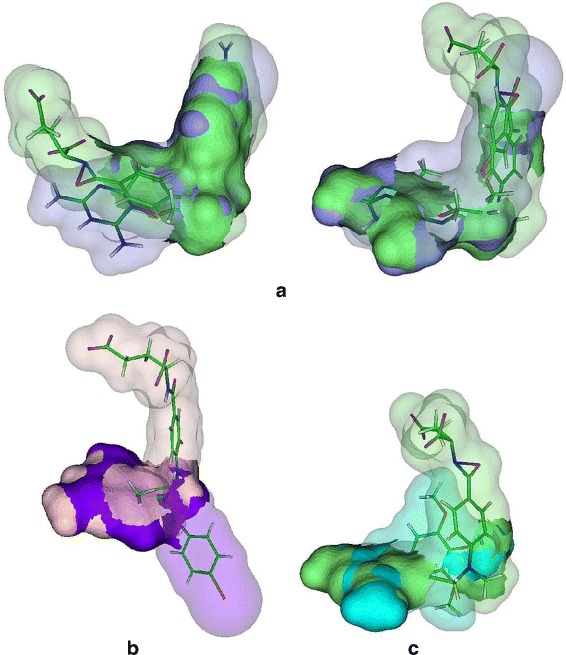
Alignments of the ligand surfaces found by MS3ALIGN and not SURFCOMP for the DHFR dataset. **a** The best two alignments MTX (green) and WRB (purple). **b** The best alignment of FOL (light pink) with WRB (purple) is along the two amino groups attached to the aromatic ring in both ligands. **c** Alignment between MTX (green) and TOP (cyan), where the two amino groups attached to an aromatic ring align

We note that SURFCOMP reports only two good alignments of FOL with MTX and WRB. We were able to recover a similar alignment of FOL and MTX. MS3ALIGN does not determine the same alignment reported by SURFCOMP for the case of FOL and WRB. Figure 11b shows the alignment determined by MS3ALIGN where the alignment is along the C =*N*−*C*= N moieties with amino groups at the two and four positions from triazine and pteridine of WRB and MTX respectively. SURFCOMP reports an alignment where the brominated benzyl group of WRB aligns with the central benzyl group of FOL which is different from the best alignment we determine.

Relevant alignments determined by MS3ALIGN for the pairs MTX-TMP, MTX-WRB, and FOL-WRB, that were not determined by SURFCOMP are shown in Fig. 11.

**Fig. 12:**
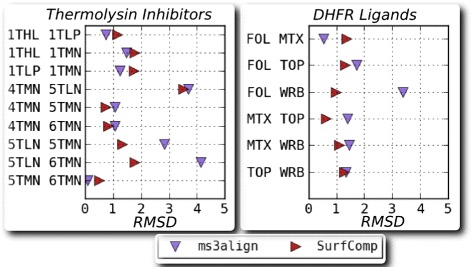
Plot of the RMS distance between ligand surfaces after alignment using MS3ALIGN and SURFCOMP. *(left)* Plot for the dataset of thermolysin inhibitor ligands. The ligands are identified by the PDB they were extracted from. *(right)* Plot for the dataset of ligands bound to DHFR enzymes. The ligands are identified by their abbreviation

Figure 12 presents the RMS distances between the pairs of surfaces after alignment using both MS3ALIGN and SURFCOMP. We note here that small RMSD values (<1.5*Å*) are indicative of successful alignments whereas larger RMSD values (1.5−5) do not necessarily indicate failure of the alignment. This is particularly true when the reported alignment is a partial alignment of surfaces where a small fraction of the surface is aligned. Table 1 tabulates and compares the aligning portions of the ligands in both datasets using MS3ALIGN and SURFCOMP. In conclusion, we were able to replicate all alignments, except one, reported by Hofbauer et al. in their evaluation of SURFCOMP. Additionally, we were able to obtain relevant alignments of similar chemical moieties.

**Table 1:** Comparison of the chemically relevant alignments of SurfComp and MS3ALIGN.

Ligand pair	SURFCOMP aligns	MS3ALIGN aligns
1THL-1TLP	Tryptophan moiety	Tryptophan moiety
1THL-1TMN	Tryptophan moiety	Benzyl moiety
		Tryptophan moiety (6th best)
1THL-3TMN	Tryptophan moiety	–
1TLP-1TMN	Tryptophan moiety	Tryptophan moiety
1TLP-3TMN	Tryptophan moiety	–
1TMN-3TMN	Tryptophan moiety	–
4TMN-5TLN	–	Benzyl moiety
4TMN-5TMN	L-Alanine moiety	L-Alanine moiety
4TMN-6TMN	L-Alanine moiety	L-LAanine moiety
5TLN-5TMN	–	Benzyl moiety
5TLN-6TMN	–	Benzyl moiety
5TMN-6TMN	4-methyl pentanoic	4-methyl pentanoic acid group
	acid group	
FOL-TOP	–	–
FOL-MTX	Entire ligands	Entire ligands
FOL-WRB	Benzyl moiety	C =*N*−*C*= N part of triazine and
		pteridine groups along with amino
		groups
TOP-MTX	–	C =*N*−*C*= N part of triazine and
		pteridine groups along with amino
		groups
TOP-WRB	–	C =*N*−*C*= N part of triazine and
		pteridine groups along with amino
		groups
MTX-WRB	–	C =*N*−*C*= N part of triazine and
		pteridine groups along with amino
		groups

The chemical structures of the ligands are presented in the Additional file 1

### A validation using POCKETMATCH and PYMOL

In this experiment, we validate alignments generated using MS3ALIGN against those generated using PYMOL [32]. Here, the objective is to study the alignments against known alignments generated using PYMOL’s “super” command. This command aligns proteins using a dynamic programming approach followed by multiple refinement cycles that improve the fit by eliminating pairings with high relative variability. We curate a dataset of thirty one protein structures from the PDB [31], each interacting with one of eight types of ligands. The dataset is chosen so that the ligands represent a range of structural variability at their site of interaction. The structural variability of the pocket is quantified using POCKETMATCH [18]. For each binding site, POCKETMATCH generates 90 sorted lists of distances from the three dimensional coordinates and chemical properties of the site. For a pair of sites, a normalized score based on the similarity of the pair of 90 lists is computed. Sites having a POCKETMATCH Pmax score greater than 0.6 are statistically shown to be structurally similar, with a score of 1 indicating identical sites. A variety of ligands ranging from small sugars such as glucose to molecules containing substituted sugars such as NDP, fatty acids such as ACD (arachidonic acid), vitamins such as Biotin (BTN) and Retinoic Acid (REA) are chosen. In the Additional file 1, we provide the details pertaining to the molecules and the ligands.

**Fig. 13:**
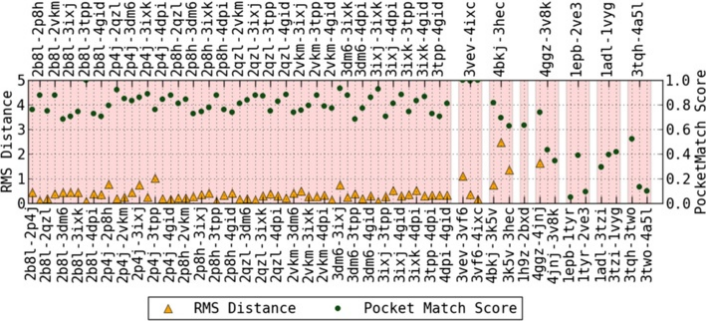
The RMS distance between pockets transformed by PYMOL’s and MS3ALIGN’s alignment. The corresponding POCKETMATCH indicate overall consensus between the different methods

**Fig. 14:**
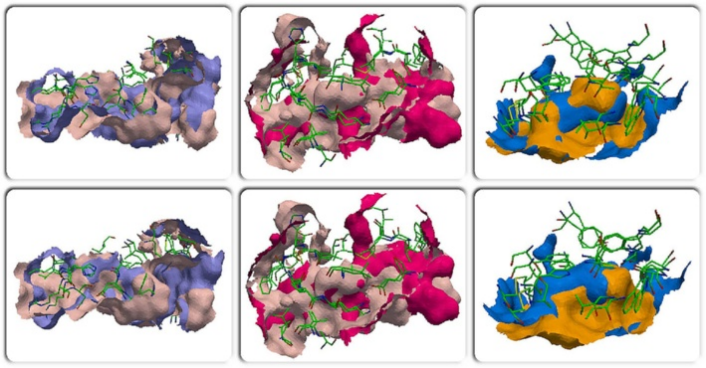
Alignments of pockets formed by the ligands STI in 4BKJ and 3HEC (*left*), STI in 3K5V and 3HEC (*middle*), and BTN in 4GGZ and 4JNJ (*right*), using MS3ALIGN (*top-row*) and PYMOL (*bottom-row*)

For this experiment, the binding surfaces of each ligand type is aligned with each other using MS3ALIGN with *R*_*c*_=1.2*Å*, *T*_*s*_=0.1, *T*_*ms*_=0.15, *T*_*mrd*_=1.2*Å*. In Fig. 13, the RMS distance between the pockets transformed by PYMOL’s and MS3ALIGN’s alignment is plotted for the set of considered pocket pairs. The POCKETMATCH score between each pair is also shown for comparison. When the RMS distance between the pockets transformed by PYMOL’s and MS3ALIGN’s alignment is less than 1.2*Å*, we conclude that both alignments are equivalent. In Fig. 14, we visually verify the validity of alignments of pairs where the RMS distance is between 1.2 and 5*Å*. We conclude that we were able to successfully determine alignments of pocket surfaces whose POCKETMATCH score is greater than 0.7 and in some cases even 0.6.

### Aligning electron microscopy iso-surfaces

In this experiment, we consider two sets of related iso-surfaces generated from cryo electron microscopy scans obtained from the EMDataBank [39]. The first dataset comprises of a related set of fragment antigen binding of HIV antibodies. The second dataset comprises of a related set of HIV antibodies. Both datasets are available at resolutions of approximately 20*Å*. Table 2 summarizes the details of the two datasets used. An iso-surface is defined as the set of points where the density value is equal to a pre-specified constant. Molecular surfaces are extracted from cryo-EM data by computing the iso-surface at a carefully chosen iso-value.

**Table 2:** Electron Microscopy datasets used in the alignment experiments.

EMDB ID	Iso-value	Imaging	Imaging box
		resolution (*Å*)	Size (*Å* ×*Å* ×*Å*)
5918	2.05	21	328×328×328
5919	2.69	19	348×348×348
5920	2.27	25	328×328×328
5323	2.2	20	410×410×410
5324	1.45	20	410×410×410
5325	2.0	20	410×410×410

The iso-value refers to the density value suggested by EMData bank for representing the molecular surface

**Fig. 15:**
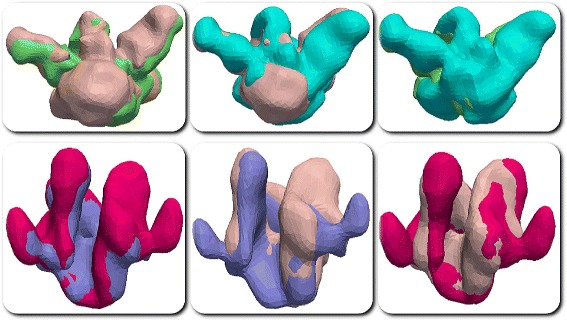
Alignments of the iso-surfaces of the cryo Electron Microscopy datasets. Pairwise alignments of the first dataset is shown in the top row and pairwise alignments of the second dataset is shown in the bottom row. Pairwise alignments between: *(top-left)* 5918 (skin pink) and 5919 (green), *(top-middle)* 5918 (skin pink) and 5920 (cyan), *(top-right)* 5919 (green) and 5920 (cyan), *(bottom-left)* 5323 (purple) and 5324 (magenta), *(bottom-middle)* 5323 (purple) and 5325 (skin pink), and *(bottom-right)* 5324 (magenta) and 5325 (skin pink)

For both datasets, we set the parameter *R*_*c*_=30*Å*, *T*_*s*_=0.05, *T*_*ms*_=0.1, *T*_*mrd*_=30*Å*, and computed alignments using MS3ALIGN. Figure 15 shows the respective pairwise alignments of the iso-surfaces of both datasets. Table 3 shows the RMS distance between pairs surfaces from the two datasets after alignment using MS3ALIGN. Here the RMSD is computed after alignment as the root mean square of the closest distance from every point of the first surface to the second surface and vice versa. Thus, we conclude that the alignments were successful.

**Table 3:** RMS distances between pairs of iso-surfaces after alignment from both datasets shown in Table 2

A	B	RMSD (*Å*)
5918	5919	6.69
5918	5920	7.00
5919	5920	8.32
5323	5324	5.71
5323	5325	8.74
5324	5325	10.75

## Conclusions

In this paper, we present a method to align molecular surfaces by identifying and establishing correspondences between significant protrusions on the surface. We also present MS3ALIGN, a tool that implements this method.

A key advantage of our method is robust segmentation of the surface into segments that can be individually evaluated for correspondences. Furthermore, due to its purely geometric design, it is applicable to molecular surfaces arising from various sources such as the PDB and Electron-Microscopy scans. This is a key advantage over existing methods such as SURFCOMP, PBSALIGN, and PYMOL, which rely on protein sequence data and other derived scalar values such as the electrostatic potential, which are often not directly available/computable. In the future, we plan to expand MS3ALIGN to align surfaces by including other geometric properties such as spherical harmonics and Zernike coefficients of individual segments. These global properties may be applied to smaller segments resulting in a method for determining alignments using a blend of local and global properties. Currently, we use only the local curvature property. Another possible direction for future work is an extension towards a framework that harmoniously incorporates additional physico-chemical properties that may be available.

## Additional file

Additional file 1 **Supplementary material for “** MS3ALIGN **: An efficient molecular surface aligner using the topology of surface curvature”.** (PDF 629 kb)

## Abbreviations

PDB: Protein data bank
DHFR: DiHydro folate reductase
