# Supplementary material for: MS3ALIGN: an efficient molecular surface aligner using the topology of surface curvature
Source: BMC Bioinformatics. 2016 Jan 12;17:26. doi: 10.1186/s12859-015-0874-8 (PMC4710026; doi:10.1186/s12859-015-0874-8)
Supplement: Supplementary file 1 — Supplementary material for “ MS3ALIGN : An efficient molecular surface aligner using the topology of surface curvature”. (PDF 629 kb) [file 12859_2015_874_MOESM1_ESM.pdf]

RESEARCH

Supplementary material for "MS3ALIGN: An efficient molecular surface aligner using the topology of surface curvature"

Nithin Shivashankar[1*], Sonali Patil[1], Amrisha Bhosle[2], Nagasuma Chandra[2] and Vijay Natarajan[3]

*Correspondence: nithin@csa.iisc.ernet.in
[1]Department of Computer Science and Automation, Indian Institute of Science, 560012 Bangalore, India
Full list of author information is available at the end of the article

Abstract

This is the supplementary material for the paper titled "MS3ALIGN*: An efficient protein surface aligner using the topology of surface curvature*". Additional figures and data pertaining to the evaluation of MS3ALIGN is presented.

Keywords: Molecular Alignments; Molecular Surfaces

Appendix 1: Dataset for performance Evaluation

In this section we present data pertaining to the set of molecules used in evaluating the performance of MS3ALIGN. Table 2 shows the set of proteins along with additional data comprising of x-ray crystallographic resolution, protein length, SCOPe 2.01 class and fold [1] (if available), and a brief secondary structure description.

PDB ID(s)	Macromolecule Name	Resolution (Å)	Length (#amino acids)	Class (SCOPe)	Fold (SCOPe)	Description
4hg7	MDM2	1.6	97	a: All alpha proteins	a.42: SWIB/MDM2 domain	4 α helices: open bundle; capped by two small 3-stranded beta-sheets
3vy6	Zymogen granule membrane protein 6	2.0	141	N/A	N/A	β-prism-fold composed of three β-sheets and each is made up of three to four β-strands
4dwn	Bcl10-interacting CARD protein	1.58	100	N/A	N/A	7 α helices
4itc	K1 domain of Lys-gingipain W83	1.55	173	N/A	N/A	β-sandwich composed of two antiparallel β-sheets
3vfz	Probable RNA polymerase sigma-D factor	1.90	86	N/A	N/A	4 α helices
4j3y, 4j44	XIAP-BIR2 domain	1.45 1.30	86	N/A	N/A	3-stranded antiparallel β-sheet surrounded by 5 α-helices
3fpu	Evasin-1	1.76	100	d: Alpha and beta proteins (a+b)	d9:IL8-like	7 β strands forming three antiparallel β sheets, one short α helix
2hpl	PNGase	1.80	100	N/A	N/A	Left-handed antiparallel four-helical bundle connected by 310 helix, β strand, a short H3 helix and a twisted antiparallel β sheet
4m6j	Dihydrofolate reductase	1.20	187	c: Alpha and beta proteins (a/b)	c71:Dihydrofolate reductase like	3 layers: a/b/a; mixed β-sheet of 8 strands, order 34251687; strand 8 is antiparallel to the rest
3wde	Probable ATP-dependent Clp protease ATP-binding subunit	1.44	153	N/A	N/A	8 α-helices. The fold of the NTD contains two repeats of a 4-helix motif
4jeg, 4je4	Tyrosine-protein phosphatase non-receptor type 11	2.30 2.31	124	d: Alpha and beta proteins (a+b)	d:93:SH2-like	3 layers: a/b/a; antiparallel β-sheet of 5 strands is flanked by two α-helices
4m6g	Peptidoglycan Amidase Rv3717	2.10	225	N/A	N/A	A central 6-stranded β-sheet, 6 surrounding α-helices
4heg, 4kb9, 4hdf, 4hdb	HIV-1 protease	1.46 1.29 1.29 1.49	99	b: All beta proteins	b.50: Acid proteases	Closed Barrel : 1 α helix and 2 antiparallel β-sheets
4hu6	General control protein GCN4	2.30	35	N/A	N/A	α helical coiled coil with 2 α helices
4kgt	Streptococcal Protein GB1 Backbone Modified Variant: Aib10, D-Pro47	2.00	55	N/A	N/A	4 stranded β sheet and 1 α helix

Table 1 Details of the 20 proteins chosen for Performance Analysis.

Appendix 2: Datasets for comparison with SurfComp

In this section, we provide the chemical structure of the datasets used to compare MS3ALIGN with SURFCOMP.

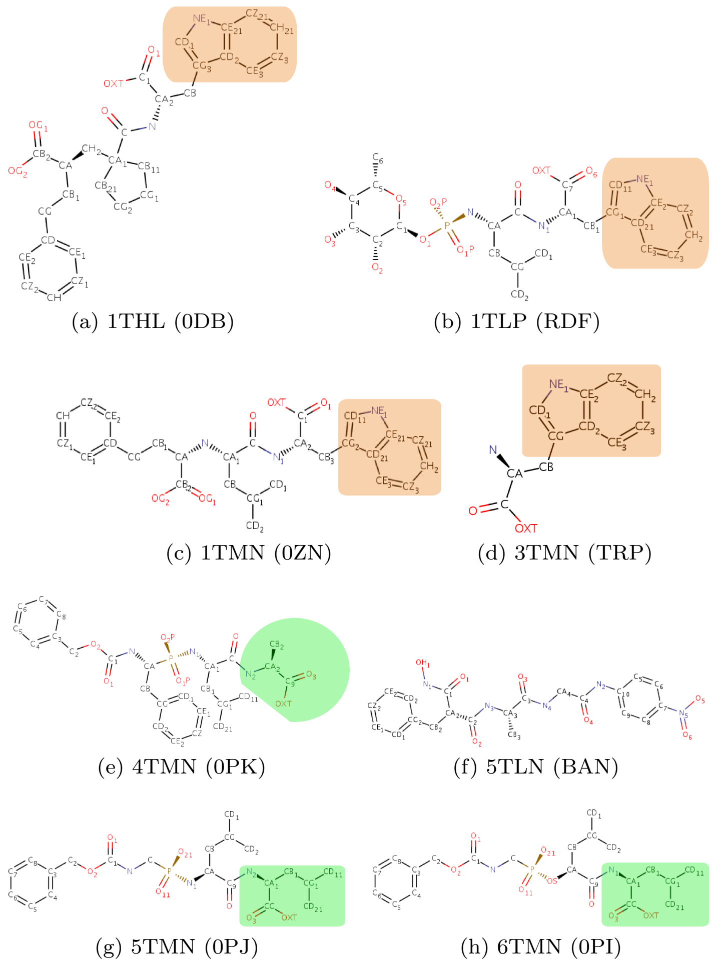

(a) 1THL (0DB) (b) 1TLP (RDF)

(c) 1TMN (0ZN) (d) 3TMN (TRP)

(e) 4TMN (0PK) (f) 5TLN (BAN)

(g) 5TMN (0PJ) (h) 6TMN (0PI)

Figure 1 Thermolysin Inhibitors. (a)-(d) Ligand structures with Tryptophan shown in the orange boxes. (e)-(h) Ligand structures with aliphatic residue shown within green shaded regions. The ligand structure images were obtained from the RCSB's [2] Protein Data Bank website.

Thermolysin Inhibitor Dataset

In this experiment, SURFCOMP computes pairwise alignments within two sets of thermolysin inhibitor ligands. Figure 1 shows the chemical structure of the ligands in both the sets.

DHFR Dataset

In this experiment, alignments of surfaces of four ligands interacting with DHFR are analyzed. In this set Folic acid (FOL) and Methotrexate (MTX) have very similar sub-structures. Trimethoprim (TOP) and Br-WR99210 (WRB) share similar structures in terms of two amino groups attached to a heterocylic ring. Similar sub-structures are present in MTX and FOL with minor differences. Figure 2 shows the chemical structures of the molecules.

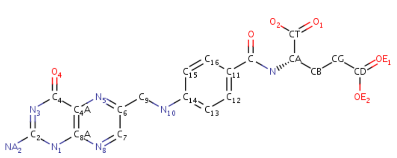

(a) Folic Acid (FOL)

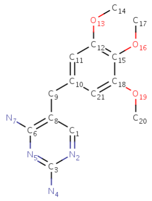

(b) Trimethoprim (TOP)

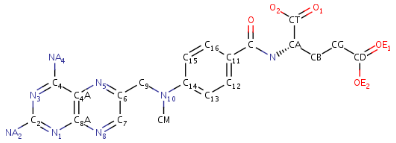

(c) Methotrexate (MTX)

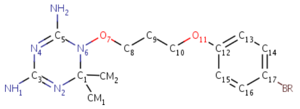

(d) Br-WR99210 (WRB)

Figure 2 DHFR ligands FOL, MTX, TOP, and WRB taken from pdbs 1DHF, 1DF7, 1DG5, and 1DG7 respectively. The ligand structure images were obtained from the RCSB's [2] Protein Data Bank website.

Appendix 3: Dataset for validation using PocketMatch and PyMol

Table 2 shows data pertaining to the molecules used in the validation of MS3ALIGN with POCKETMATCH and PYMOL.

Set No.	PDB ID(s)	Lig ID	Ligand Name	Identifier	Mol Wt (g/mol)
1	3vev, 3vf6, 4ixc	GLC	Glucose	alpha-D-glucopyranose	180.16
2	4bkj, 3k5v, 3hec	STI	Imatinib	4-[(4-methylpiperazin-1-yl)methyl]-N-{4-methyl-3-[(4-pyridin-3-ylpyrimidin-2-yl)amino]phenyl}benzamide	493.60
3	1h9z, 2bxd	RWF	Warfarin	4-hydroxy-3-[(1R)-3-oxo-1-phenylbutyl]-2H-chromen-2-one	308.33
4	4ggz, 4jnj, 3v8k	BTN	Biotin	5-[(3aS,4S,6aR)-2-oxohexahydro-1H-thieno[3,4-d]imidazol-4-yl]pentanoic acid	244.31
5	1adl, 3tzi, 1vyg	ACD	Arachidonic acid	(5Z,8Z,11Z,14Z)-icosa-5,8,11,14-tetraenoic acid	304.47
6	3tqh, 3two, 4a51	NDP	Dihydro-nicotinamide-adenine-dinucleotide phosphate	[[(2R,3S,4R,5R)-5-(3-aminocarbonyl-4H-pyridin-1-yl)-3,4-dihydroxy-oxolan-2-yl]methoxy-hydroxy-phosphoryl]	745.42
7	1epb, 1tyr, 2ve3	REA	Retinoic acid	(2E,4E,6E,8E)-3,7-dimethyl-9-(2,6,6-trimethyl-1-cyclohexenyl)nona-2,4,6,8-tetraenoic acid	300.44
8	2bl8, 3tpp	5HA	β-secretase inhibitor	N-[(1S,2R)-1-benzyl-3-(cyclopropylamino)-2-hydroxypropyl]-5-[methyl(methylsulfonyl)amino]-N'-[(1R)-1-phenylethyl]benzene-1,3-dicarboxamide	578.2
8	2g94	ZPQ	β-secretase inhibitor	N~2~-[(2R,4S,5S)-5-{[N-{[(3,5-dimethyl-1H-pyrazol-1-yl)methoxy]carbonyl}-3-(methylsulfonyl)-L-alanyl]amino}-4-hydroxy-2,7-dimethyloctanoyl]-N-(2-methylpropyl)-L-valinamide	658.85
8	3ixj	586	β-secretase inhibitor	N-{(1S,2S,4R)-5-{[(1S)-1-(benzylcarbamoyl)-2-methylpropyl]amino}-1-[(3,5-difluorophenoxy)methyl]-2-hydroxy-4-methoxy-5-oxopentyl}-5-[methyl(methylsulfonyl)amino]-N'-[(1R)-1-phenylethyl]benzene-1,3-dicarboxamide	851.96
8	3dm6	757	β-secretase inhibitor	5-[[(2S)-2-[[(3R,4S)-5-(3,5-difluorophenoxy)-3-hydroxy-4-[[3-(methyl-methylsulfonyl-amino)-5-[[(1R)-1-phenylethyl]carbamoyl]phenyl]carbonylamino]pentanoyl]amino]-3-methyl-butanoyl]amino]benzene-1,3-dicarboxylic acid	881.90
8	2p4j	23I	β-secretase inhibitor	-[(1S,2S,4R)-2-hydroxy-4-methyl-5-({(1S)-2-methyl-1-[(1-methylethyl)carbamoyl]propyl}amino)-1-(2-methylpropyl)-5-oxopentyl]-5-[methyl(methylsulfonyl)amino]-N'-[(1R)-1-phenylethyl]benzene-1,3-dicarboxamide	701.92
8	3ixk	929	β-secretase inhibitor	N-[(2S,3S,5R)-1-[(3,5-difluorophenyl)methoxy]-3-hydroxy-5-methyl-6-[[(2S)-3-methyl-1-oxo-1-(phenylmethylamino)butan-2-yl]amino]-6-oxo-hexan-2-yl]-5-(methyl-methylsulfonyl-amino)-N'-[(1R)-1-phenylethyl]benzene-1,3-dicarboxamide	849.98
8	2p8h	MY9	β-secretase inhibitor	N-{(1S,2S)-1-benzyl-2-hydroxy-2-[(4S)-1,2,2-trimethyl-5-oxoimidazolidin-4-yl]ethyl}-N'-[(1R)-1-(4-fluorophenyl)ethyl]-5-[methyl(methylsulfonyl)amino]benzene-1,3-dicarboxamide	653.76
8	2qzl	IXS	β-secretase inhibitor	N-[(1S)-1-benzyl-2-({(1S)-1-methyl-2-[(2-methylpropyl)amino]-2-oxoethyl}amino)ethyl]-N'-[(1R)-1-(4-fluorophenyl)ethyl]-5-[methyl(methylsulfonyl)amino]benzene-1,3-dicarboxamide	653.81
8	4dpi	0N1	β-secretase inhibitor	(4R,6E,11S)-16-methyl-11-[(1R)-1-oxidanyl-2-[(3-propan-2-ylphenyl)methylamino]ethyl]-4-phenyl-9-oxa-3,12-diazabicyclo[12.3.1]octadeca-1(18),6,14,16-tetraene-2,13-dione	555.71
8	2vkm	BSD	β-secretase inhibitor	N'-[(2S,3R)-3-hydroxy-4-[(3-methoxyphenyl)methylamino]-1-phenyl-butan-2-yl]-5-(methyl-methylsulfonyl-amino)-N-[(1R)-1-phenylethyl]benzene-1,3-dicarboxamide	658.81
8	4gid	0GH	β-secretase inhibitor	5-[methyl(methylsulfonyl)amino]-N1-[(2S)-1-[[(2S,3R)-1-(2-methylpropylamino)-3-oxidanyl-1-oxidanylidene-butan-2-yl]amino]-3-phenyl-propan-2-yl]-N3-[(1R)-1-phenylethyl]benzene-1,3-dicarboxamide	665.84

Table 2 Validation dataset comprising of proteins bound to eight types of ligands.

List of abbreviations used
PDB: *protein data bank*, DHFR: *DiHydro Folate Reductase*.

Competing interests
The authors declare that they have no competing interests.

Author details
[1]Department of Computer Science and Automation, Indian Institute of Science, 560012 Bangalore, India. [2]Department of Biochemistry, Indian Institute of Science, 560012 Bangalore, India. [3]Department of Computer Science and Automation, and Supercomputer Education and Research Centre, Indian Institute of Science, 560012 Bangalore, India.

References
1. Fox NK, C.J. Brenner SE: SCOPe: Structural Classification of Proteins—extended, integrating SCOP and ASTRAL data and classification of new structures. Nucleic Acids Research (2014)
2. Berman, H.M., *et al.*: The protein data bank. Nucleic Acids Res **28**, 235–242 (2000)
